# Conditional Ablation of Ezh2 in Murine Hearts Reveals Its Essential Roles in Endocardial Cushion Formation, Cardiomyocyte Proliferation and Survival

**DOI:** 10.1371/journal.pone.0031005

**Published:** 2012-02-01

**Authors:** Li Chen, Yanlin Ma, Eun Young Kim, Wei Yu, Robert J. Schwartz, Ling Qian, Jun Wang

**Affiliations:** 1 Department of Stem Cell Engineering, Basic Research Laboratories, Texas Heart Institute, Houston, Texas, United States of America; 2 Institute of Biosciences and Technology, Texas A&M Health Science Center, Houston, Texas, United States of America; 3 Program in Genes and Development, The University of Texas Health Science Center at Houston, Houston, Texas, United States of America; 4 Department of Biochemistry and Molecular Biology, University of Houston, Houston, Texas, United States of America; Northwestern University, United States of America

## Abstract

Ezh2 is a histone trimethyltransferase that silences genes mainly via catalyzing trimethylation of histone 3 lysine 27 (H3K27Me3). The role of Ezh2 as a regulator of gene silencing and cell proliferation in cancer development has been extensively investigated; however, its function in heart development during embryonic cardiogenesis has not been well studied. In the present study, we used a genetically modified mouse system in which Ezh2 was specifically ablated in the mouse heart. We identified a wide spectrum of cardiovascular malformations in the Ezh2 mutant mice, which collectively led to perinatal death. In the Ezh2 mutant heart, the endocardial cushions (ECs) were hypoplastic and the endothelial-to-mesenchymal transition (EMT) process was impaired. The hearts of Ezh2 mutant mice also exhibited decreased cardiomyocyte proliferation and increased apoptosis. We further identified that the Hey2 gene, which is important for cardiomyocyte proliferation and cardiac morphogenesis, is a downstream target of Ezh2. The regulation of Hey2 expression by Ezh2 may be independent of Notch signaling activity. Our work defines an indispensible role of the chromatin remodeling factor Ezh2 in normal cardiovascular development.

## Introduction

The development of normal cardiac structure/function is essential for the survival of mammalian embryo. Cardiac development is a complex process that proceeds through cardiac remodeling, endocardial cushion (EC) development, and outflow tract formation. Disturbance of one or more of these processes may lead to cardiac structural malformations, i.e., congenital heart defects (CHDs), which are the most common of all human birth defects [1]. EC formation, which involves endocardial cell activation, endothelial-to-mesenchymal transition (EMT), and the proliferation, migration and subsequent invasion of mesenchymal cells into the acellular matrix (the cardiac jelly) to eventually form mature and functional ECs, is a critical process associated with atrioventricular septation and outflow tract formation. Although a number of transcription factors and signaling molecules were shown to be involved in normal heart development [2], [3], [4], the genetic network underlying development of CHDs, including deficiency in EC formation, remains an issue of intense interest.

Epigenetic systems that activate/silence gene expression are emerging as a key regulator in cell fate determination, stem cell pluripotency maintenance, and normal embryogenesis. The two fundamental epigenetic systems that have been extensively studied and have been shown to regulate gene activity are the chromatin remodeling Polycomb group (PcG) proteins including the polycomb repressive complex (PRC) 1 and 2, and the Trithorax-group (TrxG) complexes including the SWI/SNF complex. The PRC mainly catalyzes trimethylation of H3 lysine 27 (H3K27Me3) for gene silencing, whereas the TrxG complexes catalyzes trimethylation of H3 lysine 4 (H3K4Me3) for activation; both groups preferentially target the histone 3 on CpG islands that are present in the *cis*-regulatory region of a broad spectrum of genes [5], [6], [7]. PRC2 contains Ezh2, the only enzymatically active subunit in PRC2 that catalyzes H3K27Me3 via its SET domain, although to complete this enzymatic reaction requires physical interaction of Ezh2 with its partners [8], [9], [10]. The trimethylation of H3K27 provides a platform to recruit PRC1, facilitating silencing of the target genes. Thus, catalysis of H3K27Me3 by Ezh2 is a critical step that enables PcG proteins to accomplish their repressive function. In addition to its well-defined suppressive activity, Ezh2 may act as a co-activator. For instance, Ezh2 potentiated target gene activity via direct physical association with the estrogen receptor and β-catenin [11], and with RelA/RelB [12]. The positive impacts exerted by Ezh2 on these target genes were independent of its histone methyltransferase activity, i.e., SET domain. In addition, Ezh2-catalyzed H3K27Me3 has also been proposed to play a role in either activating or maintaining the active status of transcription of certain genes [13]. The above findings therefore suggest that Ezh2 exhibits dual functions of either activation or inhibition, which may be context-dependent.

The role of Ezh2 as a regulator of gene silencing, cell proliferation and embryonic stem cell identity has been previously studied [14], [15]. For instance, overexpression of *Ezh2* specifically in the mammary epithelial cells promoted epithelial growth [16], whereas decrease of *Ezh2* in a numerous cell lines resulted in defective cell division [17], [18]. Ezh2 was also shown to be involved in modulation of apoptosis. For example, Ezh2 suppressed E2F1-mediated apoptosis via epigenetically repressing expression of *Bim*, a pro-apoptotic factor that was a downstream target of E2F [19]. In addition, Ezh2 was required for normal murine embryogenesis, as demonstrated by early embryonic lethality of *Ezh2^−/−^* embryos [20]. Indeed, Ezh2 contributed to the development of a number of tissues/organs, such as the development of B cells, hair follicles, and limbs [21], [22], [23]. However, whether Ezh2 has crucial functions in cardiovascular development during embryogenesis has not been well investigated, although the detection of *Ezh2* mRNA in the developing mouse embryonic heart at E9.5 [24] pointed to its potential role in cardiogenesis. This is in a sharp contrast to the well-defined roles of SWI/SNF complex components such as Baf60c and Brg1 in cardiac morphogenesis [25], [26].

In this study, we conditionally knocked out *Ezh2* in murine hearts using mice with floxed *Ezh2* alleles and *Nkx2.5-cre*, the latter which is a cardiac-specific *cre* expression line in which the cre recombinase expression is driven by the endogenous *Nkx2.5* promoter [27]. This *cre* is active in the cardiac progenitor cells and throughout the full phase of heart development. We demonstrated that homozygous knockout of *Ezh2* in mouse hearts led to lethal cardiovascular malformations with underdevelopment of the ECs. *Ezh2* mutant hearts also exhibited decreased cardiomyocyte proliferation and increased apoptosis. We further showed that *Hey2*, a member of the hairy and enhancer of split related family, was a downstream target of Ezh2, and this regulation was independent of Notch signaling activity. These observations and associated mechanisms we describe here provided novel insights into the regulation of cardiac development by the epigenetic modifier Ezh2.

## Materials and Methods

### Ethics Statement

The animals were handled in accordance with institutional guidelines with the approval (protocol ID 11006) of the Institutional Animal Care and Use Committee of the Texas A&M University Health Science Center at Houston.

### Generation of genetically modified mice

The generation and characterization of *Ezh2* floxed (*Ezh2^fl/fl^*) mice, in which the SET domain that harbors enzymatic activity was flanked by *loxP* sites, were detailed previously [23]. *Ezh2^fl/fl^* mice were crossed with *Nkx2.5-cre* mice [27] to eventually produce conditional *Ezh2* mutant *Ezh2^fl/fl^:Nkx2.5-cre+* (*Ezh2-cKO*) mice. Timed matings between *Ezh2^fl/+^:Nkx2.5-cre+* and *Ezh2^fl/fl^* mice were arranged as needed, and the day when a plug was seen was taken as E0.5. Embryos or embryonic hearts at various developmental stages such as E10.5, E12.5, E16.5, E18.5 or postnatal day (P) 1 were collected for RNA/protein analysis, or for histology. All animal procedures were performed in compliance with institutional guidelines and with approved protocol ID 11006 of the IACUC at the Texas A&M University Health Science Center at Houston.

### Antibodies

Antibodies against trimethyl-histone 3-Lys27 (H3K27Me3), Tbx2 and Sox9 were purchased from Millipore. Antibodies against Ki67 and Snail were obtained from Abcam. Monoclonal anti-cardiac Troponin T (cTnT) antibody was from Lab Vision. Anti-phospho Histone 3-Ser10 (pH 3) was purchased from Upstate, monoclonal anti-alpha-smooth muscle actin (α-SMA) from Sigma, and anti-PECAM1 antibody from BD Biosciences. Antibodies against Ezh2, NFATc1, Histone H3 (H3) and GAPDH were from Santa Cruz Biotechnology, Inc. The fluorophore-conjugated secondary antibodies Alexa Fluor® 488 (donkey anti-mouse) and 594 (goat anti-rabbit) were from Invitrogen.

### Plasmid construction, cell culture, transient transfection and western blot

Mouse *Ezh2* cDNA was PCR-amplified and inserted in frame after the HA tag of the PCGN vector via KpnI and BamHI sites (PCGN-*Ezh2*). The Notch reporter pJT123A, which contains 8× CBF binding sites [28], and the cDNA of the intracellular domain of Notch 2 (Notch2-ic) were gifts from Dr. Paul Ling (Baylor College of Medicine, Houston, USA). The Notch2-ic cDNA was then subcloned into PCGN vector after the HA tag on KpnI and BamHI sites (PCGN-hNotch2-ic). The cDNA inserts of Ezh2 and hNotch2-ic were confirmed by sequencing. The *Hey2* promoter luciferase reporter construct (*Hey2-Luc*), which contains a 1 kb fragment of the *Hey2* promoter region, was a gift from Dr. Kimberly Foreman (Loyola University Medical Center, Maywood, USA [29]). HeLa cells were maintained in Dulbecco's Modified Eagle's Medium containing 10% fetal bovine serum. Transient transfections were performed using HeLa cells cultured in 24 well plates for using Lipofectamine 2000 (Invitrogen) according to the protocol provided by the manufacturer. Luciferase activity assays were then performed as detailed previously [30], [31]. Briefly, 100 ng of *Hey2-Luc* or pJT123A was co-transfected with the empty vector as a control or with various dosages of expression vectors, as indicated in the figure legend. The empty vector was used to maintain the balance of the total amount of plasmids used in the reporter assay. Luciferase activity was measured 48 h after transfection using Monolight™ 3010 (Pharmingen). Promoter activity was expressed as the ratio of luciferase activity obtained in the presence of specific factor(s) to that of the control group with the presence of only empty vector. Luciferase activity was expressed as mean ± SEM from at least two independent assays, with each carried out in duplicate.

Western blots (WB) were performed on lysates purified from E18.5 hearts. Protein extract (120 µg) from control and *Ezh2-cKO* hearts was subjected to electrophoresis using 4–12% NuPage SDS gel (Invitrogen) and then transferred to PVDF membrane and WB performed using the antibodies indicated in the figure legend, and visualized with chemiluminescence.

### Reverse transcription-PCR (RT-PCR) and microarray assays

RNAs were extracted from wild type (WT) embryonic hearts at indicated developmental stages, or from E16.5 control (*Ezh2^fl/fl^*) and *Ezh2-cKO* hearts, using Trizol according to the manufacturer's protocol. Microarray service was provided by Phalanx Biotech (OneArray Express) with three samples per group, and each sample was analyzed in triplicate. Reverse transcription reaction was carried out using 1 µg total RNA, cloned reverse transcriptase (Invitrogen) and Oligo dT, which generated a final 50 µl volume reaction to generate the complementary DNA (cDNA). The cDNA (1 µl for Ezh2 and 0.5 µl for GAPDH, respectively) was subsequently used in semi-quantitative PCR with two alternative PCR cycles (30 or 35). qPCR was performed on machine 7900HT (Applied Biosystems) using SYBR®-Green and the gene-specific probes. The sequences of oligos used in this study are shown below: *Ezh2*: forward, 5′ ACTTACTGCTGGCACCGTCT 3′, reverse, 5′ TGCATCCACCACAAAATCAT 3′; *Ezh1*: forward, 5′ TGGTGTCTTGCAAAAACTGC 3′, reverse, 5′ AGGTTGAAGAGGAAGCTGGA 3′; *Hey2*: forward, 5′ AAGCGCCCTTGTGAGGAAAC 3′, reverse, 5′ CCCTCTCCTTTTCTTTCTTGC 3′; *GAPDH*: forward, 5′ ATGTTCCAGTATGACTCCACTCAC 3′, reverse, 5′ GAAGACACCAGTAGACTCCACGA 3′.

### Histology, alcian blue staining and immunofluorescence staining

For histological analysis, embryos or heart tissues were fixed in 10% paraformaldehyde (PFA) at the stages indicated in each figure legend, dehydrated and embedded in paraffin for preparation of histological sections at 5 or 10 µm thickness. The slides were rehydrated, followed by hematoxylin and eosin (H&E) staining according to the manufacturer's instructions. Alcian blue staining was performed according to the manufacturer's instructions.

The procedure of immunofluorescence staining was described previously [32]. Briefly, for antigen retrieval, the sections were boiled in sodium citrate buffer (10 mM, pH = 6.0), and subsequently blocked in 2% normal goat serum/PBST (1× PBS/0.05% Tween20), incubated with the primary antibody of interest at appropriate concentration, followed by fluorophore-conjugated secondary antibodies in blocking solution. TUNEL staining was conducted with ApopTag® Red *In Situ* Apoptosis Detection Kit according to the manufacturer's protocol (Millipore). Sections were mounted with Vectashield with DAPI (Vector Laboratory) and photographed under an Olympus fluorescence microscope. The number of phospho Histone H3 (pH 3) positive, Ki67 positive or TUNEL positive cells (pH 3^+^, Ki67^+^ or TUNEL^+^) was scored in at least three randomly selected fields in the left ventricle or atrioventricular cushion of E12.5 or in the atrioventricular septum of E16.5 hearts, and expressed as mean ± SEM. The number of double Tbx2 and alpha-SMA positive cells (Tbx2^+^:alpha-SMA^+^) was scored in at least three randomly selected fields in the atrioventricular cushion region of E12.5 hearts. The number of Sox9 positive cells (Sox9^+^), or Snai1 positive cells (Snai1^+^) was scored in the atrioventricular cushion region of E12.5 hearts. The data were expressed as mean ± SEM.

### Chromatin immunoprecipitation assay (ChIP)

ChIP assay was performed using the EZ-ChIP™ chromatin immunoprecipitation kit (Millipore, USA) according to manufacturer's instructions, with slight modification. Briefly, homogenized heart tissues were crosslinked with 1% formaldehyde for 20 min at room temperature, harvested, and rinsed with 1× PBS. Cell nuclei were then isolated, pelleted, and sonicated. DNA fragments were subsequently enriched by immunoprecipitation with anti-Ezh2 antibody or with IgG as a control. The precipitated genomic DNA was washed, eluted from the antibody or IgG, and crosslinks were reversed by heating at 65°C. Thereafter, the DNA was recovered by phenol/chloroform extraction followed by ethanol precipitation and washing. The purified DNA pellet was resuspended in 1×TE buffer for subsequent PCR assay using the following primers targeting the first half of CpG island upstream of the transcriptional initiation site (TIS) of the *Hey2* gene: forward, 5′ CACGCGCTAGCGGTTATTCT 3′; reverse, 5′ CACGCTGCAGCCCAGCCAG 3′.

### Statistical analysis

The unpaired Student's *t* test or Chi-square test was applied to determine statistical significance between groups when applicable and the respective p-value is shown in each figure legend. p<0.05 was defined as significant and p<0.01 as highly significant.

## Results

### Conditional deletion of *Ezh2* in murine hearts led to CHDs

**Figure 1:**
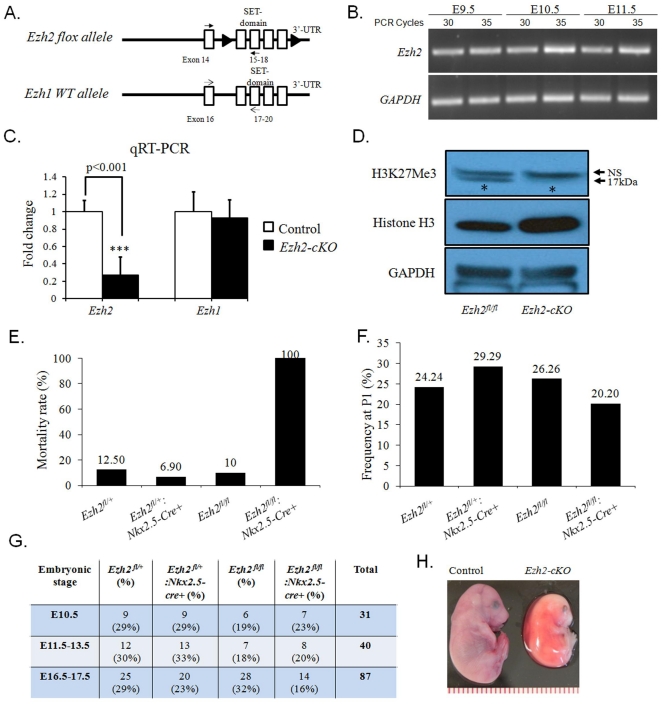
Conditional knockout of *Ezh2* gene in murine hearts caused perinatal lethality. (**A**) Schematic illustration showing the annealing sites of primer pairs of mouse *Ezh2* and *Ezh1* (a homolog of *Ezh2*). (**B**) Ezh2 expression in the murine embryonic hearts. Semi-quantitative RT-PCR with two different numbers of amplification cycles (30 and 35) was performed on RNA samples purified from WT hearts of E9.5, E10.5, and E11.5. 1 µl or 0.5 µl cDNA from reverse transcription reaction was used for detection of Ezh2 or GAPDH (as a control), respectively. (**C**) qRT-PCR analysis revealed ∼70% decrease in *Ezh2* mRNA of *Ezh2*-cKO heart (n = 3, p<0.001), but *Ezh1* mRNA was not affected, compared with that of the control littermate hearts. Data are presented as mean ± SD of 3 replicate samples for each strain/gene analyzed. (**D**) The level of H3K27Me3 was reduced in the E18.5 *Ezh2-cKO* hearts, compared to that of control. The total Histone H3 and GAPDH both serve as controls. The asterisks indicate H3K27Me3. NS, non-specific. (**E**) The neonatal mortality rate (%) and (**F**) the P1 frequency of four different genotypes resulting from the crossbreeding between *Ezh2^fl/+^:Nkx2.5-cre+* and *Ezh2^fl/fl^* mice. Data in panel E & F represent the average of 12 litters comprising a total of 99 animals. (**G**) The number and recovery rate (in parenthesis) of Ezh2^fl/+^, Ezh2^fl/+^:Nkx2.5-cre+, Ezh2^fl/fl^, and *Ezh2^fl/fl^:Nkx2.5-cre+* embryos collected at various embryonic stages are shown. Offspring from intercrossing between genotypes of *Ezh2^fl/fl^* and *Ezh2^fl/+^:Nkx2.5-cre+* mice. Note that the expected Mendelian rate of embryo recovery for each of these four genotypes is 25%. (**H**) Embryonic demise of Ezh2 mutant mice at a later gestational stage. The representative images of E16.5 WT and dead Ezh2 mutant embryos are shown.

To explore the function of Ezh2 in cardiogenesis, we first asked if Ezh2 was expressed in the developing mouse heart. Semi-quantitative RT-PCR with two different PCR cycle numbers (30 and 35) was performed on RNA samples purified from E9.5, E10.5 and E11.5 WT embryonic hearts using a pair of oligos against the SET domain of *Ezh2* (Fig. 1A, upper panel). *Ezh2* mRNA was detected as early as E9.5 (Fig. 1B), consistent with the previous report [24]. Next, *Ezh2^fl/fl^* mice were crossed with *Ezh2^fl/+^:Nkx2.5-cre+* mice to generate cardiac-specific *Ezh2* knockout (*Ezh2-cKO*) mice. To assess the deletion efficiency of *Ezh2* in heart, qRT-PCR was performed on cDNAs obtained from RNAs of E16.5 *Ezh2^fl/fl^* (control) and mutant *Ezh2-cKO* hearts using the primers mentioned above. A pair of oligos against the SET domain of *Ezh1* (Fig. 1A, lower panel), the other *Ezh* family member, was used as a control. The mRNA expression of *Ezh2* in the *Ezh2* null hearts was decreased by ∼70% relative to the control, and as expected, the expression of *Ezh1* was not affected (Fig. 1C). Correspondingly, Western blot analysis of E18.5 heart lysates prepared from control and *Ezh2* mutant mice showed a significant reduction in H3K27Me3 in *Ezh2-cKO* heart (Fig. 1D, upper panel), even in the presence of a slightly increased level of the total Histone H3 (Fig. 1D, middle panel), while no significant difference in the GAPDH level between these two groups was observed. These results indicate reduced Ezh2 function in the *Ezh2* mutant heart.

**Figure 2:**
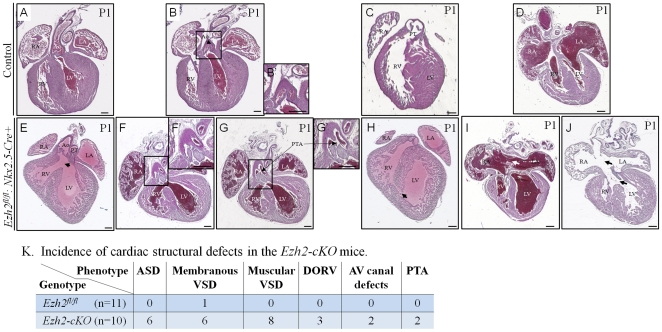
Characterization of cardiac phenotypes in *Ezh2-cKO* mice. (**A–D**) H&E staining of the coronal sections of the control mouse hearts at P1 showed normal structure of heart and great vessels. (**E–J**) H&E staining of the coronal sections of the *Ezh2-cKO* (*Ezh2^fl/fl^:Nkx2.5-cre+*) hearts at P1 showed a variety of cardiovascular defects: **E**, double outlet right ventricle (DORV) and perimembranous ventricular septal defect (VSD, arrow); **F** & **G**, two serial sections showing the transition of persistent truncus arteriosus (PTA). **F′** and **G′** are the amplification of the boxed region in **F** and **G**, respectively. Arrows in **G** and **G′** indicate the septal opening; **H**, muscular VSD (arrow); **I**, atrial septal defect (ASD, arrow); **J**, atrioventricular canal defects (AVCD, upper arrow) and membranous VSD (lower arrow). (**K**) The penetrance of cardiac defects in the mutant hearts was 100%, with variable incidence of different CHDs, as indicated. RV: right ventricle; LV, left ventricle; RA, right atrium; LA, left atrium; Ao, Aorta; PT, pulmonary trunk. n = 10. Scale bar: 100 µm.

While *Ezh2^fl/+^:Nkx2.5-cre+* mice appeared normal, viable and fertile, all *Ezh2^fl/fl^: Nkx2.5-cre+* newborn pups died before P3 (mortality rate 100%, compared with 12.50% of *Ezh2^fl/+^*, 6.90% of *Ezh2^fl/+^:Nkx2.5-cre+*, and 10% of *Ezh2^fl/fl^*, respectively, Fig. 1E), with a slight deviation of P1 frequency (20.20%) from the expected Mendelian rate of 25% (Fig. 1F). Timed matings recovered mutant embryos at a low frequency (16%) at embryonic stages between E16.5 and E17.5 (Fig. 1G), indicating embryonic loss at later gestational stages. As shown in Fig. 1H, we indeed found dead *Ezh2-cKO* embryos at E16.5. To understand the cause of death of *Ezh2* mutant mice, histological examination was performed on control (n = 11) and mutant (n = 10) hearts, and revealed 100% penetrance of cardiac defects for mutant mice, and only ∼9.1% for control (Fig. 2K, p<0.0001). A variety of cardiovascular structural malformations was observed in the mutant mice, including double outlet right ventricle (DORV, 30%, Fig. 2E), in which the aorta was connected to the right ventricle instead of the left ventricle; persistent truncus arteriosus (PTA, 20%, Fig. 2, see the transition of PTA from F to G), in which a defective septation between the pulmonary trunk and the aorta allowed a communication between these two great vessels; membranous ventricular septal defects (VSDs, 60%, Fig. 2E, F, G & J); muscular VSDs (80%, Fig. 2H); atrial septal defects (ASDs, 60%, Fig. 2I); atrioventricular canal defects (AVCD, 20%, Fig. 2J). In addition, 3 out of 10 mutant mice examined exhibited enlarged aortic valves (Fig. 2, compare F′ or G′ with B′). There was no discernible structural phenotype(s) in the ventral pharyngeal endoderm of Ezh2 mutant hearts at E10.5 (data not shown), where *Nkx2.5-cre* is also active. Collectively, these findings suggest an indispensable role of Ezh2 in normal cardiovascular morphogenesis.

### Impaired endocardial cushion development in Ezh2 mutant hearts

**Figure 3:**
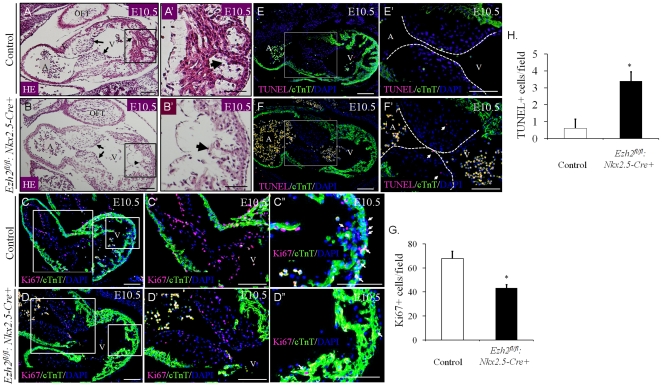
*Ezh2^fl/fl^:Nkx2.5-cre+* mice exhibited hypoplastic endocardial cushions. (**A–B′**) H&E staining revealed a decrease in the cell population in the EC region of *Ezh2* null hearts compared with that of control (arrows) at E10.5. Also, *Ezh2* mutant heart displayed defective trabeculation (arrowheads). **A′** and **B′** are the amplification of the boxed region of **A** and **B**, respectively. **A & B**, 20× magnification; **A′ & B′**, 40× magnification. (**C–D″ & G**) Double-immunofluorescence with anti-Ki67 antibody (red) and anti-cardiac Troponin T antibody (cTnT, green) revealed a reduced number of Ki67^+^ cells in both the compact myocardium and the AV cushion of *Ezh2-cKO* hearts at E10.5, compared with that of littermate controls. **C′** and **D′** are the amplification of the bigger boxed region of **C** and **D**; **C″** and **D″** are the amplification of the smaller boxed region of **C** and **D**. **C & D**, 20× magnification; **C′–D″**, 40× magnification. Scale bar, 100 µm. **G** shows that the number of Ki67^+^ cells was significantly decreased in *Ezh2-cKO* hearts relative to the control. The number of Ki67^+^ cells was counted from 10 randomly selected fields in the ECs. Data are presented as mean ± SD. *, p<0.05. n = 3 per group. (**E–F′ & H**) Double immunofluorescence of TUNEL staining (red) and anti-cTnT (green) revealed an increased number of TUNEL^+^ cells in both the AV cushion of *Ezh2-cKO* hearts at E10.5, compared with that of littermate controls. Nuclei were counterstained with DAPI, as shown in blue. **E′** and **F′** are the amplification of the boxed region of **E** and **F** respectively. **E & F**, 20× magnification; **E′& F′**, 40× magnification. Scale bar: 100 µm. **H** shows that the number of TUNEL^+^ cells was significantly increased in *Ezh2-cKO* hearts relative to the control. The number of TUNEL^+^ cells was counted from 10 randomly selected fields. Data are presented as mean ± SD. *, p<0.05. n = 3 per group. OFT, outflow tract; A, common atrium; V, common ventricle.

Normal development of the ECs is essential for the formation of outflow tract and proper septation of atria and ventricles. The presence of DORV, perimembranous VSD and AVCD in *Ezh2* mutant mice prompted us to ask whether Ezh2 regulated EC development. Since EC region in the AV canal started to develop at ∼E9.5, we first performed H&E staining on E10.5 heart sections prepared from control and *Ezh2* mutant embryos (n = 3/group) to investigate EC formation. Although the morphology of ECs in the mutant heart appeared indistinguishable from that in the control heart at this stage, it displayed substantially fewer cells in the cardiac cushions (Fig. 3A & B, arrows), indicating impaired EC development. The reduction in cell population in the EC region could be attributed to either decreased cell proliferation, or increased cell apoptosis, or both. To investigate which mechanism(s) underlied defective EC development, staining of Ki67, a cell proliferation marker, was first performed on E10.5 heart sections of both control and mutants. A considerable reduction in the cell proliferation in the EC region of *Ezh2* mutant hearts (∼40/field) compared with control hearts (∼68/field, p<0.05) was observed (Fig. 3C,D′ & G). At E10.5, the trabeculae of the *Ezh2* mutant hearts were not well formed (Fig. 3A′ & B′, arrowheads). The compact zone of mutant hearts was not significantly thinner compared with that of WT hearts at this stage; however, a significant decrease in cardiomyocyte proliferation in the compact zone of mutant hearts was already seen (Fig. 3C″ & D″, and see below). TUNEL staining performed on the same heart samples mentioned above revealed a significant elevation in apoptosis in the mutant hearts (Fig. 3E–F′ & H, n = 3/group, p<0.05). Thus, deletion of *Ezh2* was responsible for both decreased cell division and increased apoptosis in the EC region, consequently leading to hypoplastic ECs.

### Endothelial-to-mesenchymal transition (EMT) was impaired in *Ezh2* mutant hearts

**Figure 4:**
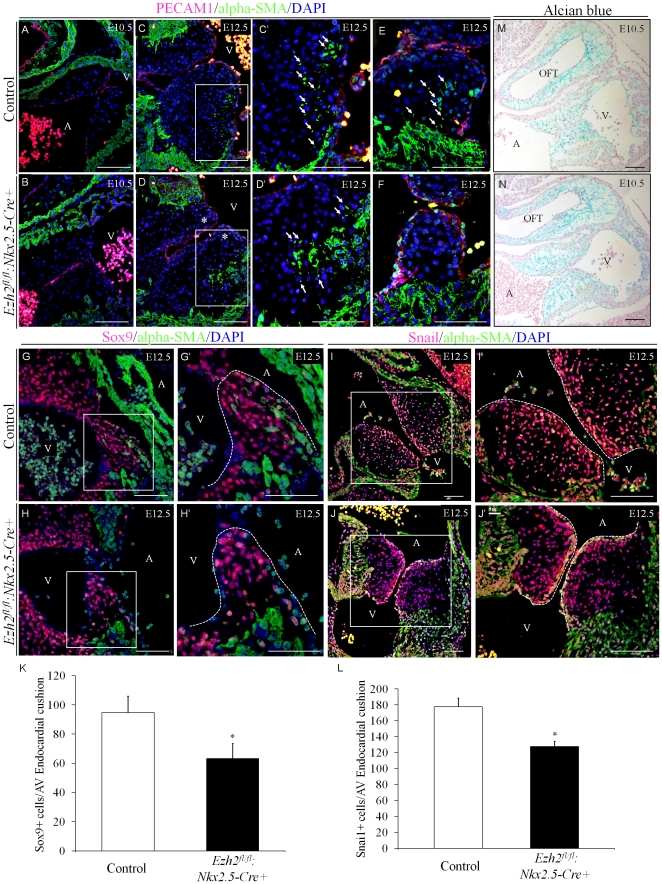
*Ezh2^fl/fl^:Nkx2.5-cre+* mice exhibited impaired endothelial-to-mesenchymal transition (EMT) in the AV cushion. (**A–F**) Double-immunofluorescence staining of the sagittal sections of E10.5–12.5 embryos with anti-PECAM1 antibody (red) and anti-α-SMA (green) revealed a reduced number of α-SMA^+^ cushion mesenchymal cells (arrows) in *Ezh2^fl/fl^:Nkx2.5-cre+* hearts compared with that of control hearts. At E10.5 and E12.5, PECAM1^+^ cells were detected lining the AV cushion, and no difference of PECAM1^+^ cells was observed between *Ezh2* mutant and control (**A–D′**). α-SMA^+^ cells were barely visible in the EC region at E10.5 in both control and mutant hearts (**A & B**), but were detected at E12.5 (**C & D′**, boxed areas). However, the number of α-SMA^+^ cells in the control EC region was significantly higher than that in the *Ezh2* mutant heart (**C′ & D′**, arrows). **C′** and **D′** are the amplification of the boxed area of **C** and **D**, respectively. Note that the cluster of α-SMA^+^ cells observed in **D′** was the migrating myocardium; only the isolated independent α-SMA^+^ cells (arrows) were EMT cells. Two additional independent sections also showed more α-SMA^+^ cells in control (**E**, arrows) than in *Ezh2^fl/fl^:Nkx2.5-cre+* (**F**). **A–D**, 20× magnification; **C′, D′, E, F**, 40× magnification. Scale bar: 100 µm. (**G–L**) Double immunofluorescence staining of the sagittal sections of E12.5 embryos with anti-Sox9 antibody (red, **G–H′**) or anti-Snai1 antibody (red, **I–J′**) and anti-alpha-SMA (green) revealed reduced number of Sox9^+^ and Snail^+^ cells in the atrioventricular endocardial cushion of *Ezh2-cKO* hearts compared with that in the littermate control hearts (n = 3). **K** and **L** show the statistical analysis of the number of Sox9^+^ and Snail^+^ cells, respectively, in the atrioventricular endocardial cushion per histological section between these two groups. Data are presented as mean ± SD. n = 3 for each group. *, p<0.05. Magnification, 20× for **G, H, I and J**, and 40× for **G′, H′, I′ and J′**. Scale bar: 100 µm. (**M–N**) Alcian blue staining in the EC extracellular matrix of E10.5 hearts showed no significant difference in morphology between *Ezh2* mutant and control hearts (n = 3/group). Magnification, 10×. Scale bar: 100 µm.

Endocardial cushions (ECs) are believed to originate from two major cell types: cardiac neural crest cells, which participate in the formation of the distal part of cardiac outflow tract, and endothelial cells, which undergo EMT and comprise ECs in the AV canal. EMT is a critical process in the EC development, and defective EMT can lead to a number of cardiac malformations such as AVCD, DORV and membranous VSD, as observed in the *Ezh2* mutant mice. To explore whether EMT was negatively affected in the *Ezh2* mutant hearts, we stained tissues for the endothelial cell marker PECAM1 and transformed mesenchymal cell marker α-SMA to detect endothelial cells or transformed mesenchymal cells in the AV canal. At E10.5, PECAM1^+^ endothelial cells lined the ECs as previously observed [33], and no significant change in the PECAM1^+^ cells between control and *Ezh2* mutant hearts was observed (Fig. 4A & B). At this stage, α-SMA+ cells were barely detected in either group. At E12.5, the pattern of PECAM1 staining in the EC region was similar to that at E10.5, but the α-SMA^+^ cells could be detected in both groups. However, the number of α-SMA^+^ cells—cushion mesenchymal cells undergoing SM differentiation—in the *Ezh2* mutant hearts was substantially lower than that in the control hearts (Fig. 4C, D, E, F, arrows), indicating defects in mesenchymal cell differentiation. To further examine the defect of EMT in the Ezh2 mutant heart, we stained for Sox9 and Snai1, both of which are transcription factors important for the EMT process [33], [34]. The number of either the Sox9^+^ (Fig. 4G, H′ & K) or the Snai1^+^ cells (Fig. 4I,J′ & L) in the ECs of *Ezh2* mutant hearts was significantly reduced by ∼30% compared with those in littermate control hearts (p<0.05, n = 3/group), further supporting that EMT was impaired.

Normal EC development requires invasion of endocardial-derived mesenchymal cells into the cardiac jelly within the AV canal, and impairments in formation of the cardiac jelly could also lead to deficient EMT. To address this issue, alcian blue staining, which can visualize acid glycosaminoglycans, such as hyaluronic acid within and surrounding the ECs, was performed on sections of embryonic hearts prepared from E10.5 *Ezh2^fl/fl^* and *Ezh2-cKO* embryos. Both control and *Ezh2* mutant hearts exhibited comparable pattern of staining (Fig. 4M & N). Thus, the cardiac jelly formation did not account for the deficient EMT seen in the *Ezh2* mutant hearts.

### *Tbx2* was downregulated in the AV myocardium of *Ezh2* mutant hearts

**Figure 5:**
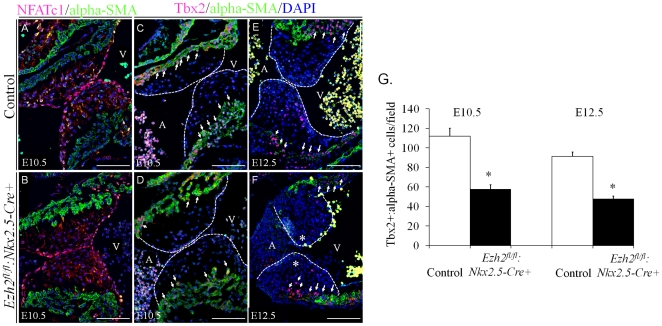
Decreased Tbx2 expression in the AV myocardium of *Ezh2* mutant mice. (**A–B**) Double immunofluorescence staining of the sagittal sections of E10.5 embryos with anti-NFATc1 antibody (red) and anti-alpha-SMA (green) revealed no change in the number of NFATc1^+^ endothelial cells in *Ezh2-cKO* hearts compared with that in littermate control hearts. (**C–F**) Double-immunofluorescence staining of the sagittal sections of E10.5–12.5 embryos with anti-Tbx2 antibody (red) and anti-alpha-SMA (green) revealed greatly reduced Tbx2^+^:α-SMA^+^ (arrows) primary myocardial cells of the atrioventricular canal in *Ezh2^fl/fl^:Nkx2.5-cre+* hearts, compared with that of the littermate control hearts. Dotted lines outlined the valve primordia. The number of Tbx2^+^:α-SMA^+^ cells was counted in three randomly selected fields of *Ezh2^fl/fl^:Nkx2.5-cre+* and control hearts of E10.5 or E12.5 embryos. Nuclei were counterstained with DAPI, as shown in blue. **G** shows the statistical analysis of Tbx2^+^:α-SMA^+^ cells between these two groups. Data are presented as mean ± SD. n = 3 for each group. *, p<0.05. Magnification, 20×. Scale bar: 100 µm.

Since the cardiac phenotypes observed in the *Ezh2-cKO* mutant mice such as defective EC formation and OFT septation were similar to those observed in mice homozygous for *Tbx2* and *NFACTc1*, both of which were shown to be important for specification and differentiation of endocardial cushion [35], [36], [37], we first examined the expression of *NFATc1* in the AV canal in both control and *Ezh2-cKO* hearts at E10.5. The staining of NFATc1 showed no significant difference between these two groups (Fig. 5A & B). We next examined *Tbx2* expression in these hearts at two developmental stages, E10.5 and E12.5, respectively. The expression of *Tbx2*, which was mainly localized in the AV myocardium of the EC region, was significantly decreased by ∼50% in the *Ezh2* mutant hearts compared with that in the control hearts at both embryonic stages examined (Fig. 5 C, D, E, F, G, arrows. n = 3/group, p<0.05). Thus, the reduction in *Tbx2* expression in the *Ezh2-cKO* heart could be a contributing factor in the development of deficient ECs, and subsequently, of cardiovascular malformation.

### Decreased cardiomyocyte proliferation and increased apoptosis in the compact and trabecular myocardium of *Ezh2* null hearts

**Figure 6:**
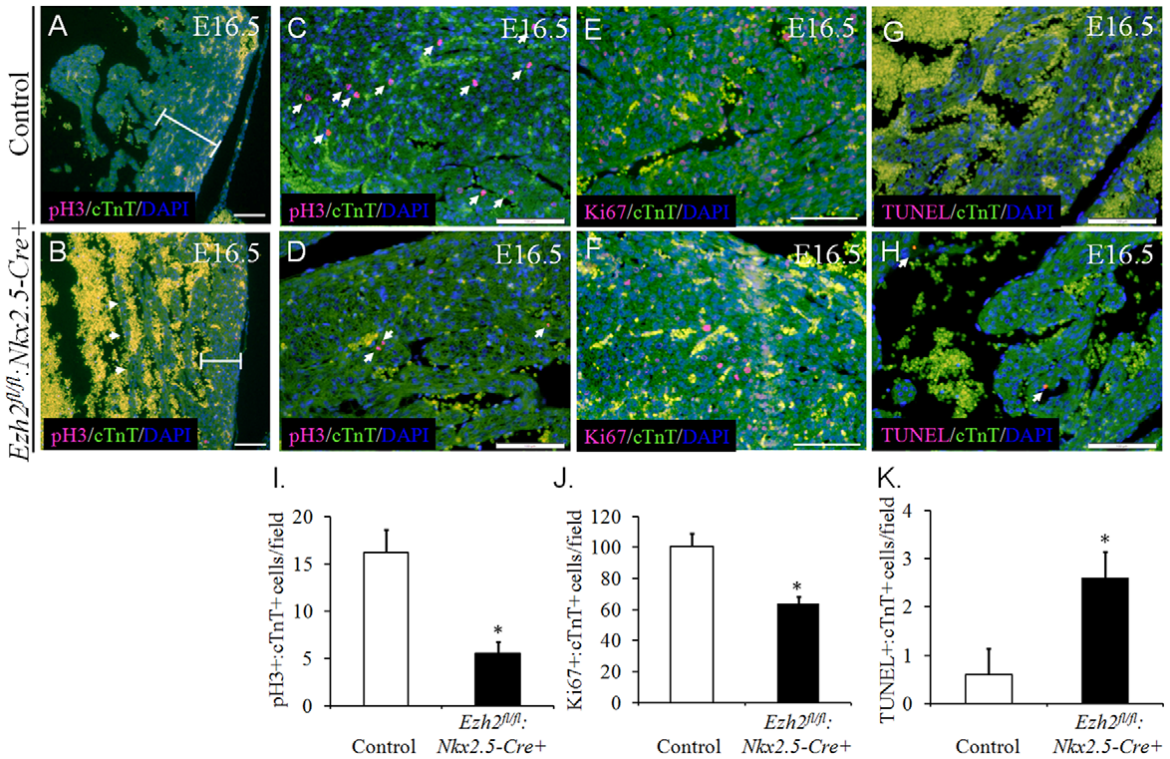
*Ezh2^fl/fl^:Nkx2.5-cre+* mice exhibited defective formation of myocardial compact zone and trabeculation. (**A–D & I**) Double immunofluorescence staining of the coronal sections of E16.5 hearts with anti-pH 3 antibody (red) and anti-cTnT antibody (green) revealed a reduced number of double pH 3^+^:cTnT^+^ cells (arrows) in *Ezh2^fl/fl^:Nkx2.5-cre+* embryos. (**A & B**) The left ventricular free wall (bar) was thinner and trabeculation (arrows) was not well formed in E16.5 *Ezh2^fl/fl^:Nkx2.5-cre+* hearts, compared with that of the littermate controls. **C & D** show **A & B** (20×) at a higher magnification (40×). **I**, the number of double pH 3^+^:cTnT^+^ cells was counted from at least three randomly selected fields of each sample and was significantly reduced in E16.5 *Ezh2^fl/fl^:Nkx2.5-cre+* hearts. n = 3/group. *, p<0.05. (**E, F & J**) Double-immunofluorescence staining of the coronal sections of E16.5 hearts with anti-Ki67 antibody (red) and anti-cTnT antibody (green) revealed a reduced number of double Ki67^+^:cTnT^+^ cells (arrows) in *Ezh2^fl/fl^:Nkx2.5-cre+* hearts relative to the littermate controls. Magnification, 20×. **J**, the number of Ki67^+^: cTnT^+^ cells were counted from at least three randomly selected fields of each sample and was significantly reduced in E16.5 *Ezh2^fl/fl^:Nkx2.5-cre+* hearts. n = 3/group. *, p<0.05. (**G, H & K**) Double-immunofluorescence analysis of TUNEL (red) and anti-cTnT antibody (green) stained coronal sections of E16.5 hearts revealed an increase in the number of double TUNEL^+^:cTnT^+^ cells (arrows) in *Ezh2^fl/fl^:Nkx2.5-cre+* hearts. Magnification, 20×. **K**, the number of TUNEL^+^:cTnT^+^ cells were counted from at least three randomly selected fields and showed significantly increased in E16.5 *Ezh2^fl/fl^:Nkx2.5-cre+* hearts. n = 3/group. *, p<0.05). Nuclei were counterstained with DAPI and shown in blue. All data were presented as means ± SD. Scale bar: 100 µm.

Given the observed decreased cardiomyocyte proliferation in the compact zone of *Ezh2-cKO* hearts at E10.5 (Fig. 3C″&D″), we further characterized the molecular phenotypes in the myocardium of *Ezh2* mutant hearts compared with that of littermate controls at a later stage, E16.5. In the mutant hearts, the trabeculae were not well defined (Fig. 6A & B, arrows), and the compact zone of the ventricular wall was thinner (Fig. 6A & B, bars), indicating defective myocardial maturation. Next, cardiomyocyte proliferation was examined by pH 3 staining. The number of cardiomyocytes entering the mitotic phase was significantly higher in control hearts (Fig. 6C & D, ∼16/field, n = 3) than in *Ezh2* mutant hearts (∼5/field, n = 3, p<0.05, Fig. 6I). Consistent with this observation, the number of total dividing cardiomyocytes was reduced in mutant hearts (Fig. 6E & F, arrows, ∼62/field, n = 3) compared with that in control hearts (∼98/field, n = 3, p<0.05, Fig. 6J). These observations further indicated that Ezh2 is an important regulator of cardiomyocyte division, in line with the reports of involvement of *Ezh2* in regulating the cell cycle in other tissues/cell lines [13], [17]. We also examined apoptosis in *Ezh2* mutant and control hearts at this stage using TUNEL assay, and observed a significant difference in the number of apoptotic cardiomyocytes between these two groups (Fig. 6G, H & K, arrows; mutant ∼2.5/field vs. control 0.5/field, n = 3/group, p<0.05). Taken together, these findings demonstrate Ezh2's essential role in cardiomyocyte proliferation and survival.

### Ezh2 regulated the expression of *Hey2* gene

**Figure 7:**
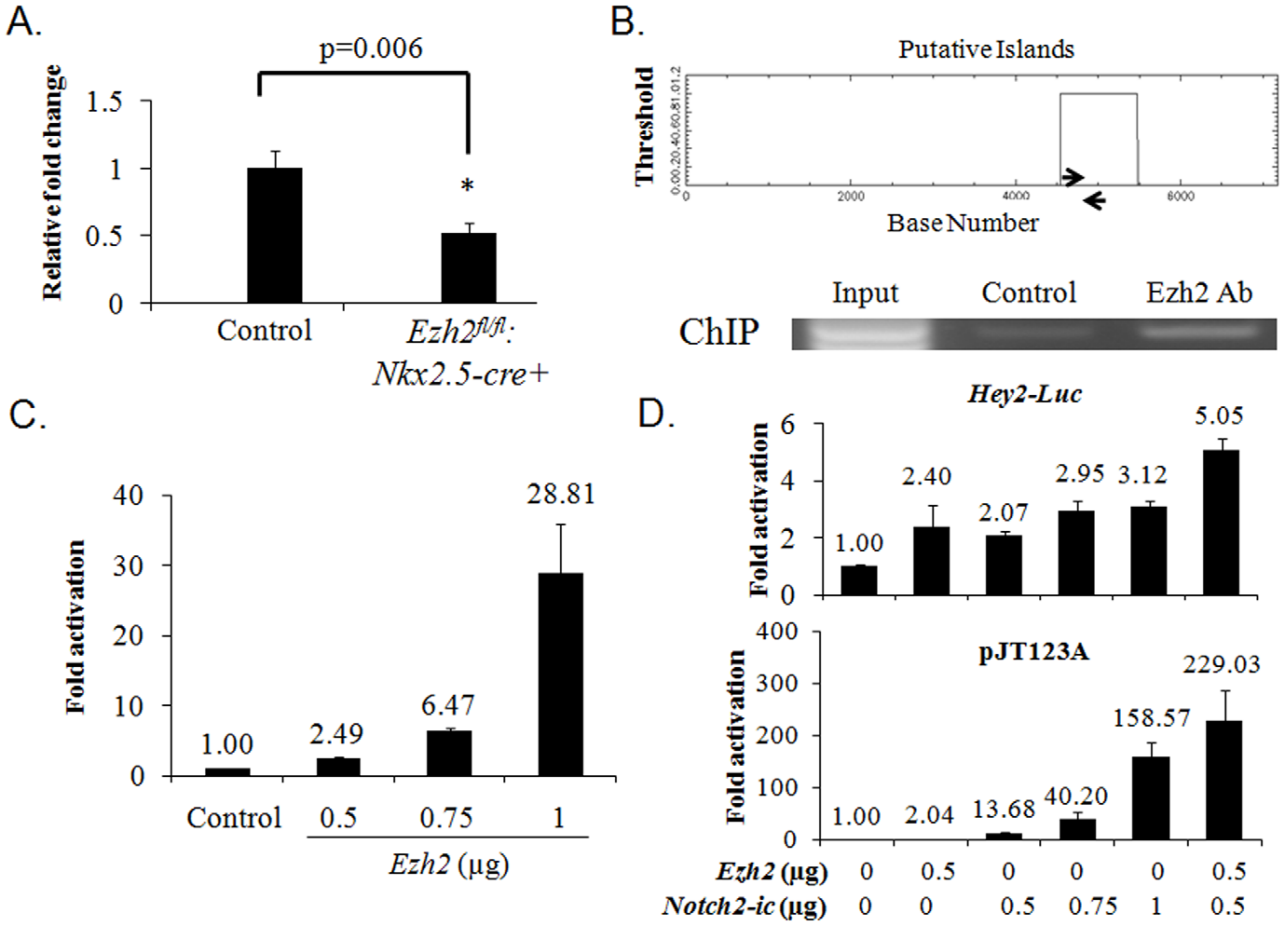
Ezh2 regulated *Hey2* expression. (**A**) RT-qPCR confirmed downregulation of *Hey2* expression in the *Ezh2* mutant hearts. n = 3 per group. Student's t-test was used for statistical analysis. (**B**) Ezh2 directly bound to the *CpG* island upstream of the *Hey2* gene. Upper panel: A putative *CpG* island was located in its 5′ cis-regulatory region (as indicated by the smaller square), spanning exon 1 of *Hey2* gene. Arrows indicate the locations of oligos for ChIP assay. Lower panel: PCR on ChIP DNA showed direct binding of Ezh2 to the *CpG* island. Pulldown with IgG served as a control. (**C**) Ezh2 potentiated *Hey2* promoter activity in a dose-dependent manner. (**D**) Functional interaction between Ezh2 and Notch in the activation of target promoters. Transfection was performed using HeLa cells as described in Materials and Methods. The doses of plasmids used in the transactivation assays are indicated. The data shown were obtained from at least two independent assays with each carried out in duplicate. The group with Ezh2 “0” and Notch2-ic “0” in **D** is the control. The number shown above each bar indicates the fold change of that particular group.

To further identify potential Ezh2-dependent genetic pathways that regulate cardiac development, we generated gene profiling data from E16.5 RNA from control and *Ezh2-cKO* heart s (n = 3/group) to look for up- or down-regulation of candidate factors critical for cardiac morphogenesis, including the EC development. In the *Ezh2* mutant hearts, there was no significant decrease in the transcription level of a number of factors that have been shown to be critical for heart development, i.e., as *Foxp1*, *Tgfb2*, *BMP2/BMP4*, *Tbx1*, *Tbx5*, *Foxm1*, *GATA4*, *5*, *6* (Table 1). However, *Hey2*, a basic helix-loop-helix (bHLH)-containing transcription factor that is required for AV canal development [38], was significantly decreased by ∼40% (p = 0.022, data not shown); the decrease in expression of *Hey2* in the *Ezh2* mutant hearts was further confirmed by quantitative RT-PCR (Fig. 7A, n = 3/group, p = 0.006). The *Hey2* mRNA level was also significantly reduced in the *Ezh2^−/−^* embryoid bodies (data not shown). The Ezh2-mediated *Hey2* expression appeared to be specific because *Hey1* and *Heyl*, the other two members of Hey family, and all other hes transcripts were expressed normally (Table 1). *Hey2* and other *Hey* family members were identified as Notch signaling targets [39], [40], [41]; however, in the *Ezh2* mutant hearts, the major components of Notch signaling pathway displayed no significant decrease in the transcription level (Table 1). Instead, *Notch4* was even significantly upregulated (Table 1). These data suggest Notch pathway-independent regulation of *Hey2* by Ezh2.

**Table 1:** No significant decrease in the expression of Notch signaling molecules and major factors that are important for cardiac development between control and *Ezh2* mutant hearts.

RefSeq	Description	Gene_symbol	fold changes	P value
NM_053202.2	forkhead box P1	Foxp1	0.959558	0.695135
NM_009367.3	transforming growth factor, beta 2	Tgfb2	1.082478	0.443397
NM_007553.2	bone morphogenetic protein 2	Bmp2	0.876828	0.463695
NM_007554.2	bone morphogenetic protein 4	Bmp4	1.335088	0.019113
NM_011532.1	T-box 1	Tbx1	0.995352	0.989775
NM_008021.4	forkhead box M1	Foxm1	0.840488	0.267564
NM_008092.3	GATA binding protein 4	Gata4	1.009269	0.920405
NM_008093.2	GATA binding protein 5	Gata5	1.158629	0.171979
NM_010258.3	GATA binding protein 6	Gata6	0.94492	0.660179
NM_011537.3	T-box 5	Tbx5	1.255253	0.353991
NM_010929.2	Notch gene homolog 4 (Drosophila)	Notch4	1.376186	0.007133
NM_010928.2	Notch gene homolog 2 (Drosophila)	Notch2	1.090368	0.865985
NM_008716.2	Notch gene homolog 3 (Drosophila)	Notch3	1.222591	0.208996
NM_008714.3	Notch gene homolog 1 (Drosophila)	Notch1	0.98534	0.922807
NM_001080928.1,NM_001080927.1,NM_009035.4	recombination signal binding protein for immunoglobulin kappa J region	Rbpj	0.929803	0.482391
NM_019454.3	delta-like 4 (Drosophila)	Dll4	1.254778	0.450871
NM_013822.4	jagged 1	Jag1	1.214836	0.495276
NM_010423.2	hairy/enhancer-of-split related with YRPW motif 1	Hey1	1.192812	0.38495
NM_013905.3	hairy/enhancer-of-split related with YRPW motif-like	Heyl	1.165378	0.431993
NM_008235.2	hairy and enhancer of split 1 (Drosophila)	Hes1	0.984655	0.941856
NM_010419.4	hairy and enhancer of split 5 (Drosophila)	Hes5	1.054865	0.517022
NM_019479.3	hairy and enhancer of split 6 (Drosophila)	Hes6	1.048307	0.713259
NM_008236.4	hairy and enhancer of split 2 (Drosophila)	Hes2	1.119306	0.789934
NM_008237.4	hairy and enhancer of split 3 (Drosophila)	Hes3	1.031301	0.766008
NM_033041.4	hairy and enhancer of split 7 (Drosophila)	Hes7	1.03991	0.775866
NM_008004.4	fibroblast growth factor 17	Fgf17	1.11111	0.653293

Microarray (OneArray Express) was performed on RNAs extracted from E16.5 control (*Ezh2^fl/fl^*) and *Ezh2-cKO* hearts. n = 3/group, with each sample carried out in triplicate.

To probe if Ezh2 could directly regulate *Hey2* expression, we revealed the presence of a *CpG* island in the 5′ flanking sequence of *Hey2* gene using a bioinformatics tool (EMBL-EBI). This *CpG* island is 932 bp, and spans exon 1 of the *Hey2* gene (Fig. 7B, upper panel, smaller box), thus rendering *Hey2* as a potential target for Ezh2. To investigate if Ezh2 could directly bind to this *CpG* island, ChIP assays were performed on WT hearts using anti-Ezh2 antibody, or as a control, IgG for pulldown; PCR was then performed using the precipitated genomic DNA (gDNA) in combination with primers designed to bind in the region flanking the first half of the *CpG* island, which is immediately upstream of the TIS of *Hey2* gene (Fig. 7B, upper panel, arrows). Robust binding of Ezh2 on the targeted region of *CpG* island was observed, but not for the control (IgG-precipitated) gDNA (Fig. 7B, lower panel). To explore if Ezh2 had any impact on the activity of the *Hey2* promoter, transactivation assays were carried out on HeLa cells transfected with the *Hey2-Luc* reporter construct, which harbors the first 455 bp of the *CpG* island, along with either an empty vector or increasing dosages of *Ezh2*. As shown in Fig. 7C, Ezh2 potentiated *Hey2-Luc* activity up to ∼28 fold in a dose-dependent fashion.

The Notch pathway is also known to positively regulate *Hey2* expression. To elucidate if there was any functional interaction between Ezh2 and Notch signaling, various dosages of the vector encoding the active domain of Notch2 (Notch2-ic) was transfected into HeLa cells with or without Ezh2. Notch2-ic activated *Hey2* promoter to a much less degree (up to ∼3 fold, Fig. 7D, upper panel) than Ezh2, and a slight synergy between Notch2-ic and Ezh2 was observed. We also tested the potential crosstalk between Ezh2 and Notch using the Notch reporter pJT123A, which contains only CBF binding sites. As expected, Notch2-ic substantially increased the activity of the Notch reporter up to ∼158 fold (Fig. 7D, lower panel), and Ezh2, at the tested dosage (0.5 µg), also activated this Notch reporter to a comparable level as Hey2-Luc. Unexpectedly, Ezh2 and Notch2-ic exerted a substantial synergistic effect on the pJT123A reporter (up to ∼229 fold at the two lowest doses of *Ezh2* and *Notch2-ic* tested). Collectively, these findings demonstrate that there is a functional interaction between Ezh2 and the Notch pathway, particularly on the tested Notch reporter.

## Discussion

As a chromatin remodeling enzyme, Ezh2 is involved in a variety of cellular events and in development of certain tissues/organs and functions by regulating target gene expression. Here we reported a particular contribution of Ezh2 to normal cardiovascular development using the approach of cardiac-specific cre-mediated ablation of *Ezh2* in murine hearts.

Our data revealed that Ezh2 is critical for the atrial and ventricular septation process and for normal formation of OFT, as demonstrated by a wide spectrum of cardiovascular anomalies observed in the *Ezh2* deficient hearts. The existence of structural malformations such as AVCD, and perimembranous VSD in the *Ezh2^fl/fl^:Nkx2.5-cre+* mice suggested defects in the EC formation, while the muscular VSD possibly resulted from decreased cardiomyocyte proliferation and/or increased apoptosis. Indeed, *Ezh2^fl/fl^:Nkx2.5-cre+* mice exhibited hypoplastic ECs with normal development of extracellular matrix. The decrease in Sox9^+^ and Snai1^+^ cells, combined with the reduction of SMA^+^ cells in the EC region of *Ezh2* mutant hearts, suggested that the transition from the endothelial phenotype to mesenchymal phenotype was not completed in the absence of *Ezh2*, and/or that the initiation of EMT was impaired in the presence of the decreased Snai1expression, given the critical role Snai1 plays in activating EMT [33]. However, the deficient EC development of *Ezh2* null hearts should be a consequence of the combined effects of decreased cell proliferation, increased apoptosis and impaired EMT, although the individual role that each of these three aspects plays in the defective EC formation remains to be dissected. It has to be pointed out that the *Ezh2* deficiency-associated cell proliferation defect and increased apoptosis observed in the EC region was not specific for ECs. Instead, it was a part of a global effect; similar alterations were also present in the other regions of the mutant hearts, such as in the ventricular walls and septum. Therefore, Ezh2 regulates cardiomyocyte proliferation and survival in different regions of the developing heart and at different stages of its development.

Although we did not investigate particularly whether the OFT cushion that derived from cardiac neural crest cells was affected in the *Ezh2^fl/fl^:Nkx2.5-cre+* mutant, the presence of DORV, PTA and defective semilunar valve leaflets of OFT indicated defective cardiac neural crest-derived mesenchyme, although these anomalies were not as prevalent as the other cardiac phenotypes. Our results were in line with the observation that Nkx2.5^+^ cardiac progenitors contributed to the formation of both the AV and OFT cushions [42]. Indeed, using *Nkx2.5-cre* to delete *BMP4* also caused defective OFT septation with impaired neural crest-derived smooth muscle [43]. Our observations pointed to the possibility that Ezh2 was present in the cardiac neural crest cells and was required for outflow tract formation. To fully understand the function of Ezh2 in the cardiac neural crest formation, specification and/or migration, alternative strategies such as the *Wnt1-cre* system, in which *Cre* recombinase expression is driven by the *Wnt1* promoter and enhancer and has often been used to conditionally delete target gene(s) in the neural crest lineage [44], [45], should be employed.

We further examined two factors that are involved in the AV canal development and outflow tract formation, Tbx2 and NFATc1, by immunostaining. We showed no significant change in NFATc1, a factor which is critical in promoting the loss of endothelial phenotype, but Tbx2 was significantly downregulated in the AV canal myocardium of the *Ezh2-cKO*. Given that the *Tbx2* knockout embryos exhibited AV canal defects and outflow tract misalignment [35], the reduction in *Tbx2* expression in the AV myocardium of *Ezh2* null mice could be a factor that facilitated development of the cardiac anomalies observed in *Ezh2-cKO* mice. Intriguingly, a significant reduction in *Tbx2* expression was only observed in the AV canal myocardium, but not in the other areas such as the ventricular wall and septum (data not shown). How Ezh2 regulates *Tbx2* expression specifically in the myocardium of AV canal remains open for further investigation.

Notch signaling is important for normal cardiac development, as evidenced by a number of studies in which mutation of Notch signaling component(s) caused cardiac defects [46]. Activation of Notch signaling elements such as Notch 4 promoted EMT [47]. In our study, we noticed a significant upregulation of Notch4 transcription in the Ezh2 mutant hearts relative to the WT controls. Despite this increase, however, EMT in the *Ezh2-cKO* hearts was not enhanced but instead was underdeveloped; this effect was opposite to the consequence of elevated Notch 4 activity. Also, a number of proposed downstream targets in the Notch signaling pathway that we examined were not significantly increased either in the *Ezh2* mutant hearts. These findings collectively suggested that 1) the increase of Notch 4 was not sufficient to significantly increase the expression of Notch target genes in the absence of Ezh2, and 2) the heart defects observed in the mutant *Ezh2^fl/fl^:Nkx2.5-cre+* mice was not due to the increased Notch signaling activity.

In the current study we identified *Hey2* as a downstream target of Ezh2. *Hey2* was highly expressed in the embryonic cardiovascular system [48], and correspondingly, knockout of *Hey2* in mice caused cardiovascular anomalies (variable phenotypes, depending upon the strain) and perinatal death; [49], [50], [51]. Hey2 was also involved in regulating apoptosis [52]. The observations associated with *Hey2* knockout included thinner ventricular wall, defects of cardiac septation and outflow tract formation, accompanied with decreased proliferation, which were similar to the defects observed in the *Ezh2* deficient murine hearts. Hey2 was proposed to have an important role in the formation of ECs [51]; however, its specific function in the EC development, i.e., EMT, cell proliferation or survival of EC region, remains to be determined. The significant decrease in the expression level of *Hey2* gene in the *Ezh2* mutant heart could be an important factor that contributed to the development of cardiovascular malformations.

Although *Hey2* was first identified together with other hairy-related bHLH transcription factors as direct targets of the Notch signaling pathway [41], a number of recent studies suggested that Notch signaling was dispensable for the regulation of *Hey2* expression in certain tissues/organs such as chick heart and the caudal presomitic mesoderm [53], [54]. A FGF signaling molecule, FGF17, also regulated *Hey2* gene expression in the pillar cells of the organ of Corti independent of Notch signaling [55]. In the *Notch1* null ventricular myocardium, the *Hey2* level was also not significantly affected [56]. Our finding of downregulation of *Hey2* expression in the *Ezh2*-deficient hearts suggested that *Hey2* was a downstream target of Ezh2 and that this regulation was independent of both Notch signaling activity and FGF17 because no significant decrease in the transcripts of Notch signaling components or FGF17 was observed in the *Ezh2* null embryonic hearts. The direct binding of Ezh2 to the *CpG* island located in the *cis*-regulatory sequence of the *Hey2* gene and activation of the *Hey2* promoter by Ezh2 raised the likelihood that Ezh2 directly regulates *Hey2* expression. Our observation that Ezh2 regulated *Hey2* gene expression adds another layer of complexity to our understanding of this pathway, at least in the developing heart. Of particular interest in the future is to probe whether Hey2 and Ezh2 can cooperatively govern cardiovascular developmental program *in vivo*.

Our work further revealed that the crosstalk between *Ezh2* and the Notch pathway existed, at least on the promoters we tested. Different levels of synergy between *Ezh2* and Notch were observed using different target promoters, indicating that the degree of crosstalk between them may be context dependent. It appears that *Ezh2* may indirectly activate the target promoter via the Notch-dependent pathway, because together with Notch2-ic, *Ezh2* elevated a Notch reporter that presumably does not have any potential *Ezh2* binding element(s). However, in *Ezh2* null mice, there was no significant change in the transcription level of most Notch downstream targets; thus, the in vivo relevance of the functional interaction between *Ezh2* and Notch signaling observed in the promoter assays remains to be clarified. It will be interesting to explore whether *Ezh1*could compensate for the loss of *Ezh2*, and thereby, cooperate with Notch to maintain the normal expression of the Notch signaling target genes. However, it is unlikely that *Ezh1* could replace the function of *Ezh2* in normal cardiac morphogenesis (see below).

Our microarray data showed that knockout of *Ezh2* in heart upregulated more genes (>600) than it downregulated (<200) (Table S1 & S2, and data not shown), which is consistent with the current belief that Ezh2 functions primarily as a repressor of target gene expression. The major function of *Ezh2* is to trimethylate H3K27. As expected, deletion of the floxed SET domain of *Ezh2* using *Nkx2.5-cre* resulted in a substantial decrease in H3K27Me3 in the heart. The residual H3K27Me3 that was still seen after *Ezh2* deletion was probably due to the presence of Ezh2 in the cells where *Nkx2.5-cre* was inactive. Alternatively, the residual H3K27Me3 could be ascribed to *Ezh1*, which was not affected by the cardiac-specific deletion of *Ezh2*. *Ezh1* was able to provide partial functional compensation for the loss of *Ezh2* in the maintenance of stem cell identity [18], however, the severe cardiovascular phenotypes and 100% immediate postnatal demise of *Ezh2^fl/fl^:Nkx2.5-cre+* mice argued that very little or no functional redundancy between Ezh1 and Ezh2 exists in the context of cardiac structural morphogenesis.

Finally, another interesting aspect is how Ezh2 activity is regulated and how that regulation affects cardiogenesis. One of the major regulatory mechanisms that modulate Ezh2 function is posttranslational modification, i.e. phosphorylation [57], [58]. More recently, Ezh2 was identified as a target of SUMO modification [59], a covalent conjugation process that is mediated by a series of enzymes and is emerging as an important regulator of cardiac development and function [60], [61]. How these modulations affect Ezh2 function, and consequently, impinge on embryonic heart development is an interesting topic to explore in the future.

## Supporting Information

Table S1 **Significantly up-regulated genes in the Ezh2 mutant hearts at E16.5 as analyzed by microarray (n = 3 for WT and Ezh2-cKO, respectively).** The threshold of fold change was set at>or = 1.5.
(XLS)

Table S2 **Significantly down-regulated genes in the Ezh2 mutant hearts at E16.5 as analyzed by microarray (n = 3 for WT and Ezh2-cKO, respectively).** The threshold of fold change was set at<or = 0.75.
(XLS)
